# Lived experiences of healthcare personnel in supporting parents around neonatal death: encounters, challenges and meaning

**DOI:** 10.1136/bmjpo-2025-004247

**Published:** 2026-04-29

**Authors:** Stuti Pant, Michael P Kelly, Maria Moreno Morales, Sudhin Thayyil, Tina Lavender

**Affiliations:** 1 Imperial College of Science Technology and Medicine, London, UK; 2 University of Cambridge, Cambridge, UK; 3 Liverpool School of Tropical Medicine, Liverpool, UK

**Keywords:** Intensive Care Units, Neonatal, Palliative Care, Qualitative research, Nursing Care, Neonatology

## Abstract

**Objective:**

To explore healthcare personnel’s (HCP) lived experiences of caring for dying newborns and supporting bereaved parents in neonatal intensive care unit (NICU), drawing on relational, ethical and organisational factors.

**Design:**

Qualitative study using hermeneutic phenomenology. Semistructured, in-depth interviews analysed thematically to interpret the meanings embedded in participants’ narratives.

**Setting:**

Three tertiary NICUs in the UK.

**Participants:**

Eight female HCP (five nurses, three doctors) with 3–20 years’ (median=12 years) NICU experience.

**Results:**

Experiences were strongly shaped by temporality and clinical trajectory. *Expected* deaths (gradual decline) enabled preparation, continuity of carer and opportunities for closeness and memory-making, whereas *unexpected* deaths (sudden deterioration) heightened moral distress, complicated communication and constrained what could be offered to families. Four themes described how optimal experiences were pursued: (1) facilitating parent–infant closeness, (2) providing comprehensive family support, (3) communication and interpersonal engagement and (4) an emotionally intense experience for HCPs.

The study revealed an overarching phenomenon—*creating an optimal experience for parents* as an implicit coping mechanism for HCP. As they cared for a dying newborn and its family, it helped them achieve a sense of accomplishment as well as the strength to power through the distress and anguish.

**Conclusions:**

The study highlights the relational foundation of neonatal end-of-life care, showing that HCPs’ well-being and quality of parental support and care provided are closely linked. Recognising this interdependence may inform training, supervision and organisational design to strengthen compassionate and sustainable NICU bereavement care.

**WHAT IS ALREADY KNOWN ON THIS TOPIC**

**WHAT THIS STUDY ADDS**

- This study interprets how neonatal healthcare personnel strive to *create an optimal experience for parents* as both a guiding practice and an implicit coping process. It details the everyday relational work—facilitating closeness, tailoring family support, maintaining consistent communication and managing emotional intensity. Uniquely this study shows how these practices and experiences differ when deaths are perceived as *expected* versus *unexpected*.

**HOW THIS STUDY MIGHT AFFECT RESEARCH, PRACTICE OR POLICY**

- Recognising relational care as central to both families’ experience and healthcare personnel’s well-being highlights the need for structured peer support, reflective practice and continuity of carer around end-of-life events. Findings can inform education, clinical guidelines and organisational policies to strengthen compassionate neonatal bereavement care within the National Health Service and similar settings.

## Introduction

 In the UK, the neonatal mortality rate is 3.0 per 1000 live births;^1^ the vast majority occur in neonatal intensive care units (NICU).^2^ Since the COVID-19 pandemic there has been an increasing recognition of mental health issues experienced by medical professionals. In neonatology, the ability of NICU healthcare personnel (HCP) to identify the need for sensitively engaging and supporting bereaved parents,^3 4^ using the appropriate language and mitigating the traumatic reactions,^5^ has long been recognised. However, newborn death is also a stressful and emotionally demanding time for HCPs themselves. They deal with ethical dilemmas and moral distress while caring for sick and terminally ill newborns and comforting grieving parents.^6 7^

In previous research HCPs, particularly nurses have reported varied experiences of moral distress, yet the emotional consequences of these experiences are often insufficiently examined.^8 9^ Despite being primarily responsible for direct bedside care, nurses typically have limited influence over treatment policy decisions, which can result in feelings of powerlessness, wavering beliefs and difficulty fulfilling their perceived advocacy role for the child.^8 10^ The impact of caring for dying neonates extends beyond immediate emotional reactions and can affect HCPs’ sense of self, shaping their values, identity and professional meaning.^11^ Quantitative studies capture the breadth of distress and burnout among neonatal staff, but lack the depth required to understand how these experiences are lived, interpreted and negotiated in practice.^12^

Existing studies describe the scope and consequences of moral distress among neonatal HCPs, but do not capture how clinicians experience, interpret and ascribe meaning to newborn death in the context of ongoing relational care, leaving a critical gap in understanding the human dimensions of neonatal end-of-life practice. Qualitative inquiry—particularly phenomenology—can illuminate the subjective dimensions of practice that are often overlooked structured surveys or metrics. Such understanding is essential both for designing effective support systems for staff and for improving the quality and consistency of bereavement care offered to parents. This study therefore explores the lived experiences of NICU HCPs in caring for newborns who die in the unit and in supporting their families through loss. The research question guiding this study was: How do NICU HCPs experience and make sense of newborn death and parental bereavement within everyday clinical practice?

### Aims

This study aimed to explore NICU HCPs’ lived experiences of newborn death and supporting grieving and bereaved parents.

## Methods

### Study design

Qualitative hermeneutic phenomenological design formed the theoretical lens to conduct the study at three tertiary level NICUs in the UK. The study followed the interpretive tradition of Martin Heidegger.^13^ Hermeneutic phenomenology allows researchers to explore the lived experiences of participants in depth, focusing on how individuals interpret and ascribe meaning to their encounters.^13 14^ It offers a methodological approach particularly suited to capturing these experiences. By exploring meaning-making processes, it enables a nuanced understanding of the interplay between clinical, relational and ethical aspects of care, including how HCPs perceive, interpret and respond to neonatal death events, providing insight into both individual and relational dimensions of care.

### Sampling

Purposive sampling was used to recruit NICU HCPs with direct experience of providing end-of-life care and supporting bereaved parents. The focus was on capturing depth and richness of data rather than data saturation. Inclusion criteria included NICU HCPs with professional experience of neonatal death. Exclusion criteria was declined consent. The sample included nurses and doctors of varying professional experience, allowing for a range of perspectives on HCPs’ experiences.

### Patient and public involvement

A patient and public involvement (PPI) group was recruited with support from Imperial College Patient Experience Research Centre and the opportunity was published on National Institute for Health and Care Research’s https://www.peopleinresearch.org/ platform to invite lived experience advisors. The group, comprising five parent advisors, contributed to study design, recruitment strategies, interview guide development and interpretation of findings. The PPI group recommended open, participant-led interviews, allowing HCPs to narrate experiences in their own words, free from predefined categories.

### Recruitment

NICU staff including doctors and nurses at the three participating National Health Service (NHS) hospitals, who had the experience of working with newborn babies who died in the NICU and supporting bereaved parents, were invited for participating in the study. Study sites were selected based on their proximity to the researcher and their capacity to capture experiences typical of tertiary-level NICUs in England. Recruitment involved initial study introduction sessions with the lead researcher (SP) for NICU teams followed by direct invitations to HCPs through email. Detailed written information was provided along with the opportunity to ask any questions regarding the study. Informed written consent was obtained and interview was scheduled for a mutually convenient time.

### Data Collection

Data were collected using semistructured, in-depth interviews between September 2021 and January 2022, with one additional interview conducted in March 2023. Due to COVID-19 restrictions, interviews were conducted online via Microsoft Teams. Interview topic guides employed broad, open-ended prompts to elicit rich narratives such as ‘Can you tell me about your experiences in providing care to newborns and families whose baby has died in the NICU?’ and later followed the parent narrative (refer online supplemental file 2-COREQ checklist).

Interviews lasted between 1 and 1.5 hours (median=1.17 hours), were audio-recorded with consent and transcribed verbatim. Member checking (participant validation) was not used; however the study findings were presented in the participating centres and were received positively. Reflexive field notes captured non-verbal cues, emotional tone and contextual insights.

### Data Analysis

Data were analysed using Van Manen’s hermeneutic phenomenological approach, incorporating holistic, selective and detailed reflection.^15^ Transcripts were read repeatedly. Significant and revealing statements were highlighted and drawn out of the transcripts, according to the selective approach suggested by Manen.^16^ These sentences were read in isolation as well as collectively with the whole text. Sentences and clusters of sentences were brought together and at this stage statements were grouped into preliminary themes, while continuing the circular move between the ‘whole’ and the ‘selective’. This process is also consistent with Heidegger’s hermeneutic circle. Using Manen’s guide to interpretation and hermeneutic circle was essential for this study,^16^ as newborn death was a multidimensional phenomenon. Therefore, it was crucial to view the data in its entirety as well as in parts, to be able to carefully examine all aspects of this experience. Broad themes were organised using thematic networks^17^ to visualise relationships. Reflexive journaling and team discussions ensured methodological rigour.

### Ethical Considerations

Given the sensitive topic, participants were reminded of their right to pause or withdraw, and interviews were scheduled for convenience. Confidentiality was maintained by removal of identifying details such as hospital identifiers or participant ethnicity details.

## Results

Eight participants (five nurses and three doctors, all female) volunteered to participate in the study and all consented. Among nurses, NICU experience ranged from 7 to 20 years; for doctors, from 3 to 13 years. Specific details of ethnicity, background as well as hospital names have not been shared to maintain participants’ anonymity.

**Figure 1:**
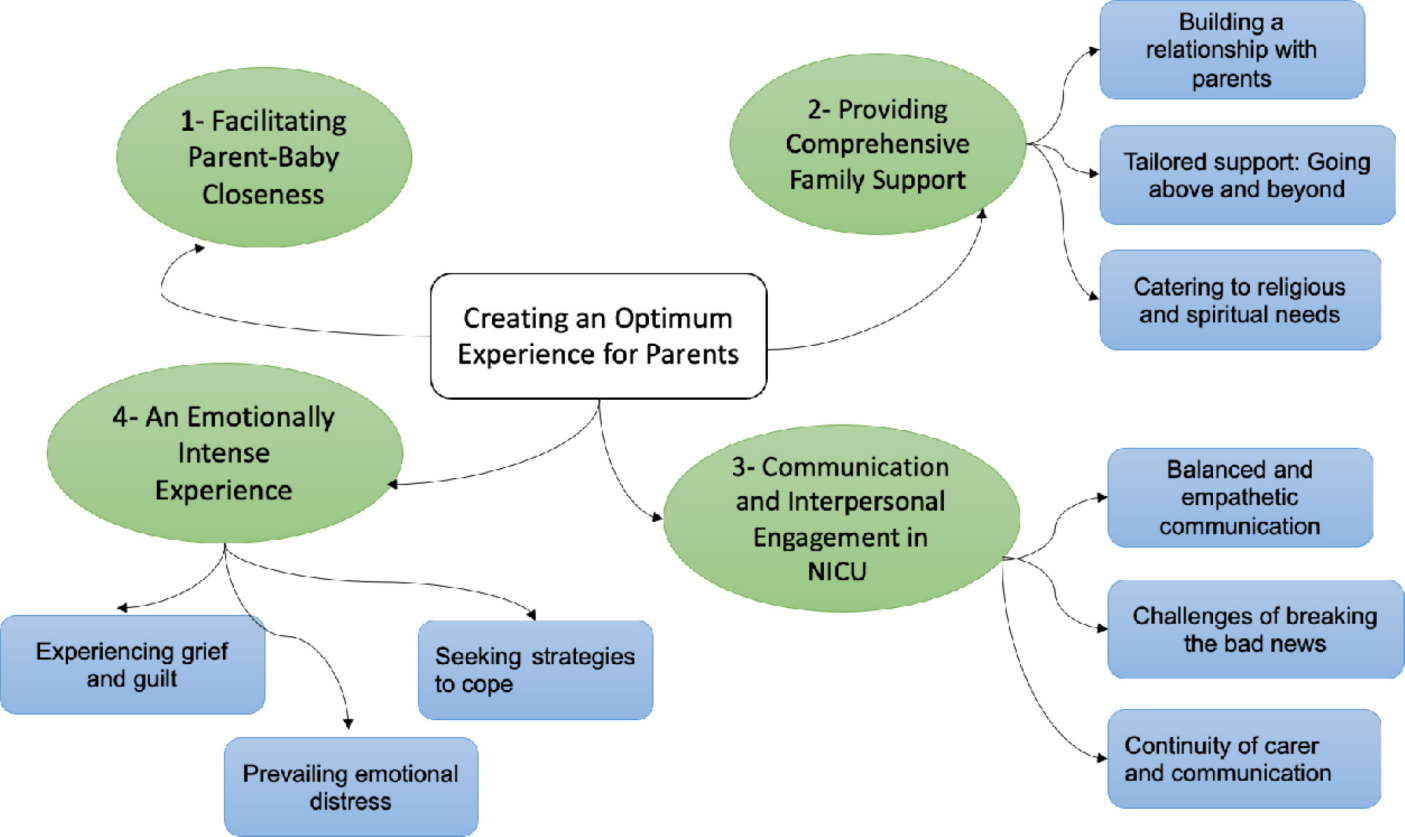
Diagrammatic representation of thematic analysis of NICU HCPs’ lived experiences. HCP, healthcare personnel; NICU, neonatal intensive care unit.

Lived experience of NICU HCPs appeared to be influenced by whether the newborn baby’s death was *‘*expected*’* (gradual decline) or *‘*unexpected’ (sudden). By *expected* deaths HCPs described being aware of the eventuality of the outcome due to their clinical experience, feeling mentally prepared for the same as well as preparing parents in time for the feared outcome. Whereas, with *unexpected* deaths where the babies deteriorated suddenly, they reported feeling caught off-guard, not allowing them time to prepare themselves as well as the parents. This temporal dimension shaped HCPs’ experiences and informed the emergent phenomenon: ‘creating an optimal experience for parents’, expressed through four interrelated themes. A diagrammatic representation of the themes is presented in figure 1 and additional participant quotations for each theme are provided in respective tables.

### Theme 1: Facilitating parent–baby closeness

HCPs viewed promoting physical and emotional closeness between parents and their baby as a central part of NICU care. Nurses described how they prioritised creating time and space for parents to hold, touch or simply sit beside their baby (table 1 Q1). They considered this process essential to helping families bond and cope with their grief. When parents were fearful or hesitant, HCPs gently encouraged contact and involvement in care activities such as washing, dressing or taking hand and foot imprints (table 1 Q2 and Q3). Memory-making was considered an important safeguard against later regret, even when parents initially declined or showed hesitance.

**Table 1:** Theme 1: facilitating parent-baby closeness—participant quotations

SNo	Illustrative quotations
Q1	*“*…whatever the parents wants to do, I don’t really care what…as long as… I can see parents having time with the baby, bonding with the baby, grieving with the baby… You know, try to support them as much as possible. I think for me that’s like more important than anything else… you try to make the baby comfortable as possible, try to change the nappy with the parents. Wash the baby with the parents… give the baby to do skin to skin with them” (Nurse 2)
Q2	*“*No, you hold their hand and touch the baby and then… they are less afraid and can hold the baby. It’s just that in the first they are afraid to touch the baby or hold. So I think…even if the baby is so sick I think the touch is really important and bringing them. You help them to face that, accept that and touch that they will become connected [with] the baby*…*” (Nurse 1)
Q3	“Yeah, it was fairly recent this happened… baby was on a ventilator so the parents agreed that we could take the baby off a ventilator, but they didn’t want to see the baby being taken off. They basically said okay, we’ll leave now and will wait in the parents room. And every now and then a relative would come and say has it happened yet, and we’d say no. And it felt… I don’t know it was very different and I think at the time… I felt sad because I thought I hope that this is actually what they want in the sense that they really don’t want to be there… after baby passed away we really kind of… not encourage but we said, you know, do you want to spend time with the baby and you could make memories and actually they’ve decided yes they did. So, they spent time with the baby afterwards, which I think was really valuable for them…” (Nurse 3)
Q4	“When you have an expected one [death],it’s like you have time to do things like you give the baby for cuddles… even if the baby is ventilated and has a lot of things… or if the grandparents want to come in and see the baby in… to wash the baby, change the nappy, things like that make the parents bond with the baby that’s… it’s beautiful. It’s beautiful to do it in a way because you are giving the opportunity for those parents… when you have that that time because time here is just precious… it’s rewarding in a way, because I mean as hard as it is at least you have done something for that family.” (Nurse 2)
Q5	“Covid… it’s been particularly difficult…made bereavement very difficult…during the first lockdown last year where we just had one parent that was allowed in every day, and but then the parents had to swap. If so, say Dad wanted to come for their mum couldn’t visit. Luckily that only lasted for a month, and we did have a bereavement in that stage. And then the parents were allowed in together, obviously. And but no extended family was. That was awful.” (Nurse 4)
Q6	“Yeah, I mean now…sometimes when I finish…whenever I had these, expected [deaths], when I finish my shift, in spite of everything I feel…that the baby at least had the parents with him and then the parents had opportunity to hold their babies for as long as they wanted to hold the baby.” (Nurse 2)

HCPs described that opportunities for closeness were easier to arrange when a baby’s death was *expected* and more difficult when deterioration was sudden or *unexpected* (table 1 Q4). COVID-19 restrictions further limited extended family’s presence and were experienced as distressing by HCPs (table 1 Q5). Supporting physical contact not only comforted parents but also gave HCPs a sense of purpose and consolation, helping them to find meaning in their work during emotionally difficult times (table 1 Q6). It brought them solace and comfort and gave them the strength to carry on.

I and my colleagues really tried the best for the baby and the parents. And even though it’s a sad situation, we can still try and find some positives…You know his life…even though sadly the baby didn’t… you know… during the time that the baby was alive to be able to show the parents’ love and care for their baby, and that baby means something and is important… is really special*.* (Nurse 3)

### Theme 2: Providing comprehensive family support

The second theme discusses HCPs’ efforts to provide comprehensive support to parents and families as a critical part of NICU care. Relational work, that is, listening, noticing and tailoring, underpinned care provided in the NICU. HCPs described supporting parents and families as a central aspect of neonatal care. Building trusting relationships was seen as essential for understanding parental needs and working together through emotionally difficult situations (table 2 Q1 and Q). Nurses, who spent the most time with families, often became key sources of comfort and continuity (table 2 Q3). They described *‘*being everything’ for the family: counsellor, advocate and practical problem-solver, while doctors emphasised aligning plans with what mattered to parents (table 2 Q4).

**Table 2:** Theme 2: providing comprehensive family support—participant quotations

SNo	Illustrative quotations
**Building a relationship with parents**	
Q1	“I think [in] NICU you form more of a relationship with the parents rather than… of course the baby is there but the baby is not talking yet so the parents are the advocates for them.” (Doctor 1)
Q2	“Parents aren’t expecting you… to come up with the right things. They just want you to listen and the dad actually said to me when I was going home, he said… I’ll have to remember that you’re human and that you have a life to go back to… And I felt really touched that in such an awful time he… in this all-consuming time his baby he said that and… even the mum during this time she said oh you need to you need to go through your lunch break… And it was like wow, like this mum is going through this like unimaginable pain and she’s thinking of me and that I need to eat… It was really touching.” (Nurse 3)
Q3	“When you’re at cot-side for 12 hours… and if you’ve got a sick baby, the parents are normally there for that length of time as well… You see them cling on to the smallest of hope that are… you’re the ones that are talking to them and be like well, how did you get the name and stuff like that just these little conversations. So, you build up this relationship very quickly with the parents that you’ve got in front of you because you’re next to them for this whole length of time, whereas the doctors are coming in perhaps every hour. But it’s the nurses that have got their relationship with them parents and who know that baby…” (Nurse 4)
Q4	“Sometimes it feels like as a nurse we are everything… we are like counsellors… sometimes I feel like you know doctor as well, multidisciplinary, cleaners just general bit of everything up and there’s so much to the day and it’s family centered care. So we’re not just caring for the baby were carrying for the family as a whole.” (Nurse 3)
Q5	“It was not in his best interest and we had to get the parents on board. Luckily even though they first went through denial in the first session, but they quickly came around and I think they realise that… there was no way forward for him, and his care was withdrawn in a garden in (hospital). I was so glad that happened but in a way… Yeah, I, I think the parents were, mum was very keen on getting him outside in the fresh air with the birds singing…she just wanted that experience for him. I was happy that it was provided for them in a very controlled way.” (Doctor 1)
Q6	“I always start with do you need like what… Who would you want for your support? because not everyone wants grandparents… Why would you offer that?” (Nurse 4)
Q7	“The most recent experience I had the family were Hindu and as it was exceptional circumstances, we allowed the extended family to come in and we also facilitated someone that they had chosen like religious figure for them to come in and sort of lead a service, before the baby passed away and so in that sense we were caring for all the family and trying to give them memories to make with the baby*.”* (Nurse 3)

NICU, neonatal intensive care unit.

Providing tailored care included small, personal gestures, negotiating extended family access (particularly during the COVID-19 pandemic) or arranging moments outdoors when possible (table 2 Q5). A precursor to individualised and tailored care was understanding what parents need and while all participants acknowledged the need for it (table 1 Q6). Institutional and policy constraints sometimes limited flexibility, but HCPs sought to maintain choice and dignity for families. Attending to religious and spiritual needs was regarded as integral to care, offering parents hope and peace (table 2 Q6).

Differences between *expected* and *unexpected* deaths shaped how support could be offered. For instance, when decline was gradual, HCPs had time to prepare parents, build rapport and plan personalised care. However, in sudden deaths, limited preparation meant interactions were more reactive and mostly focused on de-escalation and emotional containment. Overall for HCPs, being able to respond to families with empathy and creativity was important as it reinforced their sense of purpose and professional fulfilment.

We didn’t get time to prepare the family… We gave a grim picture to this family… and we asked them what their wishes were, so Dad said he wanted… He was very angry… as if it’s my fault… but I knew they were in, they are very stressed so I didn’t take any of these things personally, but I was purposely standing like a bit away from him so that because his posture was kind of aggressive (Nurse 5).

### Theme 3: Communication and interpersonal engagement

HCPs described communication with parents as one of the most demanding aspects of neonatal care. Conversations required a fine balance between honesty and empathy and careful judgement on HCPs’ part about how much information to share particularly as parents were perceived to hold on to specific words (table 3 Q1 and Q2). They worked to balance realism with compassion, ensuring families understood their baby’s condition without losing hope.

**Table 3:** Theme 3: communication and interpersonal engagement in NICU—participant quotations

SNO	Illustrative quotations
Q1	*“*We’re just trying to support… Whenever they ask something, you try to be as clear and as truthful as possible, even if it’s uncomfortable for them to hear because if they ask you, will he ever be able to eat on his own? And the doctor has said, probably not… you try to be as truthful as possible, but at the end you cannot…you don’t want to make them feel guilty for anything.” (Nurse 2)
Q2	“as a nurse as well, often all the questions get directed at you, but some questions I feel I can answer, but often I think I’ll direct them to the medical team because… These kind of circumstances parents will hold on to everything you say, and that’s quite a big pressure because you think if I say the wrong thing or something that could be misconstrued that will be remembered forever for them. And so it was really about kind of being open but also…not kind of… not giving too much away and kind of directing those sort of questions to the doctors to answer because that’s kind of their role, and we’re here to kind of care, care for the baby, provide the nursing care and support for families.” (Nurse 3)
Q3	“so if you are going to… breaking of bad news today there is a plan that the consultants will say okay at two o’clock today we’re going to sit down with the parents and then we need to discuss about the direction of care and as the baby that is very sick and dying and prepare the parents that this is the plan of care so with that… so you so if that is your allocation for the day, so you are going to prepare yourself, prepare your patient and prepare the rooms… because the first thing they will speak is the nurse when they come in because the doctors are doing something, so you kind of alert or tell them that the baby has deteriorated last night*…*” (Nurse 1)
Q4	“Yeah, so because the nature of the job is we work shift base right? So in a week they’ll meet so many people from different consultants, certainly different registrars in different SHOs. And one of the things that they would not intentionally, but they would ask everyone who’s come to see the baby and say you know, how’s this baby doing? So they ll ask the day team and then they’ll ask the night team again and even saying things like oh, you know I think he’s doing alright or he’s doing the same. Uh, they can interpret it really differently, so I feel like in such babies where you know, you know that clinically, you’re just buying the parents some time that there’s no way that this baby is going to survive, then even saying things like he’s stable or he’s OK is something that they can take very differently from what you intended to me.” (Doctor 3)
Q5	“I felt it was quite privileged to be able to look after the baby then, and I was actually allocated to different patient, but I asked to be to look after this baby because I built that rapport with the parents and I felt it was important to have that continuation and they had that trust with me and I felt that rapport and also as a professional as well almost to kind of… let the baby go as well, yeah.…” (Nurse 3)
Q6	“I had a 25 week baby who was really poorly… Mum was really poorly…she’d had to be moved to the surgical high dependency unit… The father of the baby and granddad were on the unit, so the consultant went and spoke to the parents and said, you know, your baby is incredibly sick but just yesterday I had a 24 and 25 weeker that went home…painted a lot of hope… So then that consultant went home for the day and we had another consultant…for the night shift…this was now a male consultant and he came up to me and on ward rounds, so he’s making a plan for the next two hours 4 hours and…said to me this baby is not for chest compressions… It’s not for any medications, so if the babies to deteriorate we will change the breathing tube only. And I turned around said to this consultant and I said, you cannot make that decision without speaking to the parents… I said…the previous consultant had just painted the picture that this baby is going home…so we cannot change the… how as medical team can we make the decision that actually we’re not going to do anything if this baby deteriorates? And I can say I was quite junior at this time, so we then had to go down to the surgical high dependency unit to see the family to basically tell them that their baby was really sick. And he wasn’t sure if their baby was going to make the night. Now that was a completely different conversation to, we’d had two hours later. However, it was the right conversation we should have had two hours earlier. that baby died within 24 hours.” (Nurse 4)

NICU, neonatal intensive care unit.

One of these things that I try not to be is very, very melancholy around the patients, and also try not to be very void of showing any emotions as well at the same time… you want to be realistic in in the baby’s prognosis, but at the same time… you don’t want them to become so disheartened that they just give up altogether and just not want to fight anymore*.* (Doctor 2)

Breaking bad news was described as especially challenging. When deterioration was gradual, HCPs could prepare families and plan conversations (table 3 Q3); when death was sudden, discussions were unplanned and emotionally charged. Nurses often faced parents immediately after deterioration and helped prepare families and the clinical environment before doctors led formal discussions. Staff described occasions when parental distress escalated into anger or mistrust towards the team, which they experienced as personally difficult, yet they aimed to remain calm, emotionally prepared and sensitive to each family’s background, while validating distress even when conversations became confrontational.

It was extreme premature baby and that they… it wasn’t in baby’s best interest to stay on the ventilator and the baby wouldn’t be able to survive without it. And the mum, there wasn’t a dad in the picture. Then Mum was very, very angry and she accused us of… being a business and that we basically wanted to kill her baby so we could get other babies in… And that was really hard to hear because it was… a very intense conversation. I was there as a sort of… almost like I think it made me feel sad because it made me feel like… At the time I thought. Oh God, so are we not… are we not doing a good job at this? Am I not?… my job’s never been described as just being something of a business like it just felt everything that the NHS wasn’t and… but I think it was also just a… I felt okay every parent is so different… accept and also validate their feelings*.* (Nurse 3)

HCPs highlighted how differing messages between shifts could confuse or distress families, reinforcing the value of maintaining familiar caregivers during end-of-life care (table 3 Q4, Q5 and Q6). Clear, compassionate and coordinated communication was seen as fundamental to trust, parental understanding and the creation of an optimal experience for families.

### Theme 4: An emotionally intense experience

HCPs described neonatal death as an emotionally taxing experience. Many developed bonds with babies and families and felt grief, guilt and self-doubt, especially after sudden deterioration or when parents could not be present (table 4 Q1,Q2). Nurses often felt the burden of shouldering bedside end-of-life care and to ensure the baby was cared for in the final moments and died with dignity (table 4 Q3). Several nurses also requested greater presence and engagement from doctors during the bereavement journey of parents to share emotional labour.

**Table 4:** Theme 4: an emotionally intense experience—participant quotation

SNO	Illustrative quotations
Q1	“when the baby that I was looking after died so unexpectedly. And I was the only one…I was alone preparing the baby. That was really down… that was really hard for me…preparing, dressing the baby, getting the tubes out and getting the hair and you know*…*” (Nurse 2)
Q2	“It has not affected me professionally…emotionally I struggled to pass ITU [Intensive therapy unit/NICU] I’m really glad that I am not in ITU this week to pass and see that cot space empty. I am really glad and I’ve heard other nurses say it’s hard to… It’s hard to pass and see his cot space empty. Or to see another mother-baby in it you know. So I think people are going through the emotions, all of us.” (Doctor 2)
Q3	“I think when you’ve done the end-of-life care, so once you’ve end, once you’ve stopped treatment… We then bathe the baby…do handprints and footprints. We take photos. You know that’s when it’s just the nurses, doctors don’t do any of this… Once the doctor have had that conversation that perhaps we’re stopping everything. It’s then the nurses that do it all. The only thing the doctor at, the only thing that sounds awful what the doctor has to do is to come and listen into the baby to see if it’s it’s passed or like when the baby has died. By that time you’ve been in a room with a parent in their most worst grief. Trying to make… joining your head to make sense of everything but trying to support the parents at the same time, trying to give them memories with the bath, the photos, dressing the baby… You know that’s hard and not all nurses can do that. And you can’t teach how to do that.” (Nurse 4)
Q4	“You have to go to those deliveries, and you have to tell these parents these are the statistics, and of course for them even if you give one percent or two percent, they want to take it, especially if a mother has had so many miscarriages they want to try… It’s very tricky… and sometimes they do survive, and I feel… if they survive and if they are in intensive care, you are doing this whole lot of procedures on them and we don’t know what their pain threshold is, what they are… what the babies are going through, and what they will have when they grow up, what kind of experiences they may have… We’ve had in Paediatrics, we’ve had certain preterm babies who have grown up and who come to us and they’re literally scared of needles, and they have really low pain threshold, and it’s all because of the NICU experience and we don’t know enough of what these babies experience. So yeah, I think that’s bit of a problem.” (Doctor 1)
Q5	“He was a 34 weeker,… We tried to have talks about redirection of care to the point where parents refused to speak to the medical team. They came in three times a week, stayed for less than half an hour and that was all they visited. In their head they wanted him to get to a term baby because they thought when he got to term everything would be OK. It wasn’t. We ended up withdrawing on this baby, aware, but, but only when they said so. So we kept this baby alive for eight weeks. It was incredibly hard. This was the hardest thing a, as the nursing team we’ve ever done because we were the ones caring for that baby. We would change his nappy or we would read him stories. I made sure that when I was on shift I had half an hour cuddle time… And that was just their (parent’s) coping mechanism, wasn’t it? They had a date in mind. So eventually when it did come to withdrawing a treatment… they turned said we are ready to do it today… the nursing team lead it … mum and dad had cuddles. Then mum and Dad went home… And then it was up to us as nurses to sit with him until he took his last breath and that’s what we did. We took it in turns. I did an hour. My friend did an hour… I stayed till 11:00 o’clock at night. Because for me I wasn’t going to leave until I knew the end… And so, I think as nurses and doctors are very differently made up, doctors are very medicalized nurses are very… more I don’t know more person, I don’t know more personal*…”* (Nurse 4)
Q6	“[In] sudden death… you question yourself, I’ve done everything… I feel like guilty I should have not left or blame someone like why you do some procedure without me and then… Because you know that baby is your responsibility… you’re like assessing what happened… which is really bad… you feel bad because as I said you know the mom will come…you will call them up, mummy, could you please come…I want to let you know that the baby… your baby had deteriorated and suddenly and is now on the ventilator…and when they come you know they look at you in front of your face asking what happened…” (Nurse 1)
Q7	“We didn’t get time to prepare the family… We gave a grim picture to this family… and we asked them what their wishes were, so Dad said he wanted… He was very angry…as if it’s my fault… but I knew they were in, they are very stressed so I didn’t take any of these things personally, but I was purposely standing like a bit away from him so that because his posture was kind of aggressive and I was worried that he might lose control and he might do something so it was a bit scary, but we don’t want him to left alone either, so we just we just… I just standing back with good distance so that he can’t reach me in case if he get very angry*.”* (Nurse 5)
Q8	“Yes, they do this kind of after the death they do kind of meetings so that they can get support from the psychologist… Do I go for those meetings? it’s very rarely I go. I think at the beginning when I was very new to neonatology I used to go. I never felt it’s kind of supporting. Just asking the same thing you’re going through…talking about the same thing over and over again… I don’t know about others, I maybe I’m kind of introverted person. I’m not very outspoken. I just pray to God and that’s keeping me keep going.” (Nurse 5)
Q9	“I think it’s difficult to come back the next day, but then you also see the babies that get better and that go home absolutely fine after a very long journey in the NICU. That’s very satisfying” (Nurse 2)
Q10	*“…*And even if things don’t come out the way that you wanted it, like God understands that you have tried as best as you can within or like your capacity and resources.” (Doctor 3)
Q11	“Another thing which helps to cope probably is, I don’t know it’s there… If it is very stressful day like you know someone…a genuine person come and say give you a hug like it’s OK, we can go through together. You know something like that. It’s more than enough…” (Nurse 5)
Q12	“I mean, when you have when you have a person there guiding you or supporting you, even whatever experience you have because you can be very experienced and then maybe because… your personal situation I think also influences your mood and how you are on the shift… you are human… you cannot put that aside and sometimes these things hits you harder than others. And I think the person, the team around you, not only the nurse in charge, the team around, your colleagues and everything that makes a lot of difference on how you process and how you handle situation.” (Nurse 2)

NICU, neonatal intensive care unit.

It would be good to have more support sometimes for nurses because we are just… you know they (doctors) come in and out and we are there and… but it’s quite a lot and our role entails so much. And obviously a doctor’s role is very busy too… But I think it is very nurse lead which actually for such a big thing is… it’s quite a lot. So… I think maybe more doctor involvement… (Nurse 3)

HCPs reflected on moral distress that arose when prolonged treatment seemed burdensome or when clinical views and parental wishes diverged. The eventual decision to withdraw treatment was often described as parent-led, leaving staff to manage the prolonged emotional labour of care and, at times, to accompany the baby at the point of death. These experiences intensified feelings of moral distress, self-questioning and emotional exhaustion (table 4 Q4 and Q5).

As with other themes, experiences differed by context. With *expected* deaths, HCPs could prepare themselves and parents. With *unexpected* deaths, parental reactions were sharper, conversations were unplanned and HCPs were more vulnerable to self-questioning and guilt (table 4 Q6 and Q7). They described feeling sadness, anger and grieving in a manner that was unique to themselves. It got more challenging when they were a parent themselves, because there was relatability.

We have sadness and sometimes anger of why this is happening to these babies and… I think it’s the personal process that you go through with every death because you think you will get used to it but you don’t… For me now, it’s harder actually after I have a baby, I thought it wouldn’t make a difference at the beginning, but it does… So you kind of imagine what the parents are feeling, but you cannot imagine their feelings right? (Nurse 2)

Coping strategies varied for everyone. Overall, none of the HCPs reported using the NHS counselling service provided (table 4 Q8). Preferred coping strategies included meaning-making through *‘*doing everything possible’ for the family, spirituality, small rituals (using the baby’s name, telling stories) and drawing strength from other babies who recover and get discharged home (table 4 Q9 and Q10). For most participants, having a supportive team and colleagues was the most effective means of coping in the NICU. Junior staff looked for guidance from their seniors to discuss their experience and challenges faced. It was reassuring for them to know that they have the support from the senior staff as they learnt to navigate the challenges of bereavement and newborn death (table 4 Q11 and Q12).

## Discussion

This study explored the lived experiences of NICU HCPs in caring for dying newborns and supporting bereaved parents. The hermeneutic phenomenological lens allowed interpretation of how HCPs make sense of death, transform distress into purpose and sustain professional identity. The study’s central phenomenon, ‘creating an optimal experience for parents’, reflects a dual function: fulfilling ethical and relational duties to families while sustaining HCP’s own emotional equilibrium. In participants’ accounts, relational care was not an ‘add-on’ to clinical work; it was a key way of coping, preserving confidence and continuing to provide care in the face of repeated loss.

While recent qualitative reviews have aimed to offer a more holistic understanding of providers’ experiences, they tend to summarise common categories (caring, communication, coping)^18^ rather than unpack the lived, practice-based processes through. Our study therefore adds depth by clarifying how clinicians interpret events in real time, or how relational actions become sources of meaning and endurance.

Facilitating closeness remained foundational to HCPs to ensure family-centred care, aligning with evidence that parent–infant contact supports bonding and infant development.^19 20^ Participants described enabling closeness (eg, presence, touch, skin-to-skin) and memory-making despite clinical and environmental constraints. This experience was emotionally protective for staff, because it allowed them to leave families with something meaningful despite the outcome. This interpretation extends existing accounts of neonatal end-of-life care by showing that ‘supporting parents’ is also a key mechanism through which HCPs sustain compassion and professional purpose.

Communication and tailored family support were central. HCPs emphasised learning families’ values, preferences and support networks, consistent with the principles of Family Integrated Care.^21 22^ They also emphasised continuity and consistency of messaging, reflecting prior work showing that conflicting information amplifies parental stress.^23 24^ Our findings add nuance by making visible how this plays out in routine practice: nurses frequently absorbed parents’ follow-up questions after medical conversations and described pressure to remain open while avoiding inadvertent false hope. This highlights role-boundary work as part of bereavement care, and helps explain why staff regarded language and consistency as high stakes.

Our findings build on previous work that has often centred ethical dilemmas and decision-making.^8^ HCPs reported personal grief, moral distress and emotional strain, consistent with earlier research.^25^,^27^ Importantly, distress was not limited to bereavement itself; it was also linked to situations where staff felt constrained in providing the type of relational care they believed families needed. Coping relied predominantly on peer support, senior containment/mentorship, personal reflection and spirituality, while formal counselling was seldom used or was perceived as unhelpful. This aligns with reviews calling for better targeted informal and formal support and suggests that interventions should prioritise embedded, team-based and reflective mechanisms rather than rely solely on referral to generic services.

A distinctive contribution of this study is the role of temporality. HCPs’ experiences differed when deaths were believed to be *expected* versus *unexpected*. Expected deaths allowed preparation, structured communication, continuity of carer and opportunities for closeness and memory-making, while unexpected deaths intensified moral distress and complicated communication. While previously mentioned studies do identify common sources of distress and coping, this trajectory-based difference is less clearly articulated and offers a useful lens for understanding when relational care is most feasible and when staff vulnerability to guilt and self-doubt may increase.

A novel contribution of this study is the interpretation of *creating an optimal experience for parents* as an implicit coping mechanism for NICU HCPs as they lived through newborn death in the NICU. Interpreted together, participants described deriving meaning, professional steadiness and emotional endurance through attending to parents’ relational and emotional needs, including facilitating closeness, continuity and supportive memory-making. Conversely, when opportunities to provide these experiences were constrained, HCPs were vulnerable to guilt and self-doubt. This interpretation underscores the inseparability of clinical, relational and emotional labour in NICU practice and relational engagement as both an ethical duty to families and a psychological support for staff.

### Implications for practice

A trajectory-sensitive approach to bereavement care is warranted, as highlighted by this study, the feasibility of relational support differs between expected and unexpected deaths. When decline is anticipated, teams can plan proactively for continuity of carer, structured communication, parent–baby closeness and memory-making. However, when deterioration is sudden, NICUs may benefit from a rapid, coordinated response that prioritises immediate parental support, clear role allocation for communication and timely emotional containment for families and staff. Training should therefore move beyond generic bereavement communication and use scenario-based teaching for both trajectories, including managing bedside follow-up questions, balancing honesty and hope and responding to anger or mistrust. Staff support should also reflect the finding that creating an optimal experience for parents can function as an implicit coping mechanism for HCPs: protecting time for relational care, embedding brief team reflection after deaths (particularly unexpected deaths) and ensuring visible senior support may strengthen both staff well-being and the quality and consistency of care provided to bereaved families.

### Strengths and Limitations

A key strength is the focus on newborn death as lived and experienced, rather than restricting analysis to a single component such as withdrawal decisions.^8^,^10^ The hermeneutic phenomenological approach enabled an open exploration of relational and emotional dimensions. Including both nurses and doctors adds value in a literature where professional roles can be unevenly represented.^8 25^ Sampling across three tertiary NICUs increased contextual variation compared with single-site work.

Limitations include voluntary participation and an all-female sample; male HCP perspectives were not captured, and the smaller number of doctors may mean the analysis reflects nursing experiences more strongly. As with interpretive qualitative research, findings are not intended to be statistically generalisable; transferability depends on contextual fit. The study has been shared within NHS Trust dissemination activities where the themes were recognised by NICU staff, but broader sampling across different unit types and professional groups would strengthen future work.

## Conclusion

This study illuminates NICU HCPs’ experiences during neonatal death and their efforts to support bereaved parents. Creating an optimal experience for parents emerged as both a guiding principle and an implicit coping mechanism, allowing HCPs to navigate complex clinical, relational and ethical challenges. Facilitating parent–baby closeness, providing tailored support, engaging in empathetic communication and managing emotional intensity were central to this process. Temporality and the nature of death further shaped experiences, influencing preparedness, moral distress and the ability to provide meaningful parental support. Recognising and supporting this phenomenon has significant implications for training, policy and organisational culture, highlighting strategies to simultaneously address relational care and HCPs’ well-being in the NICU.

## Supplementary material

10.1136/bmjpo-2025-004247 online supplemental file 1

10.1136/bmjpo-2025-004247 online supplemental file 2

## Data Availability

Data are available upon reasonable request.
