# Supplementary material for: Lived experiences of healthcare personnel in supporting parents around neonatal death: encounters, challenges and meaning
Source: BMJ Paediatr Open. 2026 Apr 29;10(1):e004247. doi: 10.1136/bmjpo-2025-004247 (PMC13141010; doi:10.1136/bmjpo-2025-004247)
Supplement: online supplemental file 2 [file bmjpo-10-1-s002.docx]

Supplementary File 1: COREQ checklist

Consolidated criteria for reporting qualitative studies (COREQ): 32-item checklist

Developed from:

Tong A, Sainsbury P, Craig J. Consolidated criteria for reporting qualitative research (COREQ): a 32-item checklist for interviews and focus groups. International Journal for Quality in Health Care. 2007. Volume 19, Number 6: pp. 349 – 357

| **Item No** | | **Guide Questions/Description** | **Reported on Page #** |  |
| --- | --- | --- | --- | --- |
| **Domain 1: Research team and reflexivity** | | | |  |
| **Personal Characteristics** | | | |  |
| 1. Interviewer/ facilitator | | SP (first author) conducted all interviews with close supervision from senior authors (MK, ST and TL) | Pg 10 |  |
| 2. Credentials | | PhD | Pg 1 |  |
| 3. Occupation | | Post-doctoral fellow | Pg 10 |  |
| 4. Gender | | Female | This checklist |  |
| 5. Experience and training | | SP (first author) is a social scientist and a trained qualitative researcher with extensive experience of conducting in-depth studies. The research team comprised of neonatal academics as well as senior social scientists. Senior author (TL) is an established academic with over a decade experience of conducting and supervising hermeneutic qualitative studies. | This checklist |  |
| **Relationship with participants** | | | |  |
| 6. Relationship established | | Yes, through introductory conversations, depending of participant comfort level | This checklist |  |
| 7. Participant knowledge of the interviewer | | Participants were made aware of the motivations behind the research and the perceived importance of this issue. It was reciprocated | This checklist |  |
| 8. Interviewer characteristics | | SP is not a clinician, which helped approaching the issue from a non-medical perspective. However, hermeneutic phenomenology acknowledges the role of researcher subjectivity and it was addressed during the course of research using reflexivity exercises. | This checklist |  |
| **Domain 2: study design** | | |  |  |
| **Theoretical framework** | | |  |  |
| 9. Methodological orientation and Theory | Hermeneutic phenomenology | Pg 4 |  |  |
| **Participant selection** | | |  |  |
| 10. Sampling | Purposive Sampling | Pg 4 |  |  |
| 11. Method of approach | Site visits | Pg 5 |  |  |
| 12. Sample size | 8 | Pg 5 |  |  |
| 13. Non-participation Setting | NA | Pg 6 |  |  |
| 14. Setting of data collection | MS teams | Pg 5 |  |  |
| 15. Presence of nonparticipants | No | This checklist |  |  |
| 16. Description of sample | Omitted due to risk of identification | NA |  |  |
| **Data collection** | | |  | No |
| 17. Interview guide | Topics/questions to be explored potentially/depending on the flow of conversation-  - Can you tell me about your experiences in providing care to newborns and families whose baby has died in the NICU?  Prompts, if necessary  - Can you tell me about your experience of working with parents under stress specifically with regards to such difficult decisions?  - Can you tell me if you feel prepared to handle these situations?  - Can you please tell me what happens when a baby dies?  - Can you tell me about the kind of bereavement and/or follow-up support that is offered to parents?  - Can you tell me more about your relationships with parents?  - Have you ever felt any impact of such experiences in your personal life or health?  - Can you tell me about the support you receive at your work-place  - Is there anything that would change, that would help you provide better support for bereaved parents in your NICU? | Pg 5 and this checklist |  |  |
| 18. Repeat interviews | No | This checklist |  |  |
| 19. Audio/visual recording | Audio | Pg.5 |  |  |
| 20. Field notes | Yes | Pg.5 |  |  |
| 21. Duration | 1-1.5hours | Pg 5 |  |  |
| 22. Data saturation | N/A in phenomenology | This checklist |  |  |
| 23. Transcripts returned | No | This checklist |  |  |
| **Domain 3: analysis and findings** | | |  |  |
| **Data analysis** | | |  |  |
| 24. Number of data coders | SP, reviewed by TL and MK | Pg10 |  |  |
| 25. Description of the coding tree | Coding of data was an iterative process based on the hermeneutic circle by Heidegger. Analysis was conducted in a circular fashion where data was interpreted parts of the text in relation to the whole. Therefore, there was no formal coding tree. | This checklist |  |  |
| 26. Derivation of themes | Derived and interpreted from the data | Pg 5 |  |  |
| 27. Software | None used. | This checklist |  |  |
| 28. Participant checking | Anonymised study results presented during NICU grand rounds and study presentations at the participating centres | Pg 9 |  |  |
| **Reporting** | | |  |  |
| 29. Quotations presented | Yes | Pg 6-8 , supplementary table |  |  |
| 30. Data and findings consistent | Yes | Pg 6-8 |  |  |
| 31. Clarity of major themes | yes | Pg 6-8 |  |  |
| 32. Clarity of minor themes | yes | Pg 6-8 |  |  |
