# Supplementary material for: Lived experiences of healthcare personnel in supporting parents around neonatal death: encounters, challenges and meaning
Source: BMJ Paediatr Open. 2026 Apr 29;10(1):e004247. doi: 10.1136/bmjpo-2025-004247 (PMC13141010; doi:10.1136/bmjpo-2025-004247)
Supplement: Supplementary file 3 [file bmjpo-2025-004247.reviewer_comments.pdf]

PEER REVIEW HISTORY

BMJ Paediatrics Open publishes all reviews undertaken for accepted manuscripts. Reviewers are asked to complete a checklist review form and are provided with free text boxes to elaborate on their assessment. These free text comments are reproduced below.

ARTICLE DETAILS

**TITLE (PROVISIONAL)**	Lived Experiences of Healthcare Personnel in Supporting Parents Around Neonatal Death: Encounters, Challenges, and Meaning
**AUTHORS**	Pant, Stuti<br>Kelly, Michael P<br>Morales, Maria Moreno<br>Thayyil, Sudhin<br>Lavender, Tina

VERSION 1 – REVIEW

**REVIEWER NAME**	Garstang, Joanna
**REVIEWER AFFILIATION**	University of Birmingham College of Medical and Dental Sciences, School of Nursing and Midwifery
**REVIEWER CONFLICT OF INTEREST**	No Conflict of Interest
**AI DISCLOSURE**	No, I have not used AI
**DATE REVIEW RETURNED**	18-Dec-2025

**GENERAL COMMENTS**	This is an interesting and generally well written paper, however I think the findings could be better presented. In many places they come across as superficial which I'm sure is not the case. I realise that there is a tight word count for medical journals which makes presentation of qualitative work difficult. Having so many long quotes in a supplementary table means they are not visible and are unlikely to be read. I suggest putting some of them in a table per theme, numbering the quotes and clearly referencing in the text which quote illustrates the point you are trying to make. It would make it more readable to have slightly fewer quotes too. Many of the quotes in the table do not seem to clearly fit with the themes, referencing them to the text would help with this.<br>Introduction<br>This section needs expansion to include more details on what previous studies have found. You need to detail what is already known as well as what is not known. The relevant key findings of studies ref 8-14 (para 2 of introduction) need to be presented.<br>Please state clearly what the research question was<br>Methods<br>Some further information on the methods is needed.<br>Please summarise the topic guide (this could be in a table to save words).<br>What was the background of interviewer and the research team?<br>Were transcripts checked with interviewees?<br>Did PPI group include bereaved families?<br><br>Results<br>As in my general comments these need more detail and quotes to really demonstrate the depth and richness of the data.<br>How many different NNUs did participants come from?

	Please provide some more detail of the coding structure so that it is apparent that the themes are grounded in the data<br>Is there more data on the differences between nurses' and doctors' experiences?<br>Nurses wanted greater presence of doctors. Was there data on doctors' views on that?<br>In the discussion you refer to HCPs experiencing grief, moral distress and emotional strain – this didn't really come across in your results (other than in the table)– it would be good to highlight these.<br>Discussion<br>The discussion seems to mainly summarise the findings from the results. There needs to be more information on how your results build on other research, the similarities and differences. Is there any other research that supports your finding of creating an optimal experience? Or the differences with expected vs unexpected deaths. This other research may relate to other bereavement care.<br>The implications need further expansion – the suggestions for training, staffing and debrief are fairly standard practice already. The strengths and limitations should include strengths and limitations with respect to other research.

**REVIEWER NAME**	Das, Manoja
**REVIEWER AFFILIATION**	The INCLEN Trust International, Public Health
**REVIEWER CONFLICT OF INTEREST**	No Conflict of Interest
**AI DISCLOSURE**	No, I have not used AI
**DATE REVIEW RETURNED**	21-Dec-2025

**GENERAL COMMENTS**	The manuscript presents the experiences and challenges of HCPs in sick newborn care. Some suggestions for consideration/addition are<br>There 3: The narration could check and mention if there conflicts between the different HCPs (doctors and nurses) and/or preferences for some nurses/doctors by parents for seeking counseling.<br>Theme 4: Could check and present the emotional conflicts between the reality and parent expectations and handling situations with disagreement.<br>Could check for the specific training and support received and needed for improving the coping strategies by HCPs in such situations.

VERSION 1 – AUTHOR RESPONSE

#1. This is an interesting and generally well written paper, however I think the findings could be better presented. In many places they come across as superficial which I'm sure is not the case. I realise that there is a tight word count for medical journals which makes presentation of qualitative work difficult. Having so many long quotes in a supplementary table means they are not visible and are unlikely to be read. I suggest putting some of them in a table per theme, numbering the quotes and clearly referencing in the text which quote illustrates the point you are trying to make. It would make it more readable to have slightly fewer quotes too. Many of the quotes in the table do not seem to clearly fit with the themes, referencing them to the text would help with this.

We thank the reviewer for these comments and the constructive review.
As suggested, we have now added a table for every theme with specific relevant illustrated quotations marked in text for ease of reference. We have also made edits to the results section to address the reviewers concern of coming across as superficial and to make sure the essence of the study is being communicated.

#2. Introduction

This section needs expansion to include more details on what previous studies have found. You need to detail what is already known as well as what is not known. The relevant key findings of studies ref 8-14 (para 2 of introduction) need to be presented. Please state clearly what the research question was

We thank the reviewer for this comment. We have elaborated the introduction section to provide a more details on key findings of the relevant background literature. We have also added the research question at the end of the introduction paragraph.

Please see page 4.

#3. Methods

Some further information on the methods is needed.

Please summarise the topic guide (this could be in a table to save words). What was the background of interviewer and the research team?

Were transcripts checked with interviewees?
Did PPI group include bereaved families?.

Details on background of reviewer and research team have been provided in the COREQ checklist shared as part of supplementary uploads. Information on member-checking (participant validation) was mentioned in the checklist. However, we have now incorporated this suggestion in the main text to provide more clarity to the readers (please see page 5).

We have now added the topic guide as part of the COREQ checklist and

Information on recruitment of PPI group is now added in the main text in methods section (please see page 5).

#4. Results

As in my general comments these need more detail and quotes to really demonstrate the depth and richness of the data.

How many different NNUs did participants come from?

Please provide some more detail of the coding structure so that it is apparent that the themes are grounded in the data

We thank the reviewer for this constructive feedback. We have added more details to the result section as an attempt to demonstrate the richness of the data.

There were three tertiary level NICUs that participated in the study. Thank you for pointing out, this detail was accidently missed from the paper and now has been added in the Methods-Recruitment section (please see page 5).

Coding of data was an iterative process based on the hermeneutic circle by Heidegger. No

specialist software was used in this process and the analysis was done manually to retain the essence of hermeneutic reflection. In this study, analysis was conducted in a circular fashion where data was interpreted as parts of the text in relation to the whole. Therefore, there was no formal coding tree. We have elaborated the text in Data Analysis section to give more insight into the analysis process(page 5). We have also provided a supplementary table that presents a sample of data extract from transcript of Nurse 1 interview and presents the process of breaking down meaning from data.

#5 Is there more data on the differences between nurses' and doctors' experiences? Nurses wanted greater presence of doctors. Was there data on doctors' views on that?

We have now added more details on the expectations of nurses from doctors in the main text of Theme 4 (page 8) rather than the supplementary table. As the study used purposive sampling, and not theoretical saturation, data was not analysed concurrently. Therefore, we did not use data from previously conducted interviews to support new data generation and accordingly ask questions. It would have been a good insight to gather from doctors regarding this experience shared by nurses, however no such data was generated from doctors' interviews, who mostly referred to the bereavement support as a collective effort.

#6In the discussion you refer to HCPs experiencing grief, moral distress and emotional strain – this didn't really come across in your results (other than in the table)– it would be good to highlight these..

We have added this in the results section with illustrative quotes presented in the supporting table 4. The quotations were very long and hence could not be added in the main text (please see page 8)

#7. Discussion

The discussion seems to mainly summarise the findings from the results. There needs to be more information on how your results build on other research, the similarities and differences. Is there any other research that supports your finding of creating an optimal experience? Or the differences with expected vs unexpected deaths. This other research may relate to other bereavement care.

We have edited the discussion section to incorporate feedback provided (please see page 9 and 10).
Creating an optimal experience for parents in NICU has been well established by previous research. However, the novel contribution made by this study is how this endeavour is a coping mechanism for neonatal HCPs. Similar the role of temporality and trajectory of deaths- expected vs unexpected deterioration is a unique contribution of this study and to the best of our knowledge have not been presented before.

#8. The implications need further expansion – the suggestions for training, staffing and debrief are fairly standard practice already. The strengths and limitations should include strengths and limitations with respect to other research..

Thank you for the feedback. We made edited these sections (please see page 10 and 11).

Reviewer: 2

#1. The manuscript presents the experiences and challenges of HCPs in sick newborn care. Some suggestions for consideration/addition are

Theme 3: The narration could check and mention if there conflicts between the different HCPs (doctors and nurses) and/or preferences for some nurses/doctors by parents for seeking counseling.

We thank the reviewer for these comments. There were no data reflecting conflicts between doctors and nurses. The only aspect shared in similar light was from nurses expecting more support and involvement from doctors, which has been reported in theme 4 of the results section (please see page 8).
Mostly HCPs did not prefer enrolling in the counselling services provided by the NHS as they felt it was not helpful. They preferred relying on more informal sources of support and had their distinct coping mechanisms. Unfortunately, due to space restrictions we are unable to share them separately but we have tried to summarise in our best capacity.

#2. Theme 4: Could check and present the emotional conflicts between the reality and parent expectations and handling situations with disagreement.

Could check for the specific training and support received and needed for improving the coping strategies by HCPs in such situations.

We have elaborated this part in the theme 4 and provided illustrative quotes in the supporting table (please see page 8).

#3. Could check for the specific training and support received and needed for improving the coping strategies by HCPs in such situations.

Most HCPs relied on their individual and personal coping mechanisms to deal with newborn death. Peer support in the NICU was one common thread across most participants and it was valued by all while trying to manoeuvre the grief experienced. The current formal support provided by NHS was not perceived as helpful by most HCPs and hence we have made a recommendation to strengthen peer-peer support in the units and embedding brief team reflections especially after unexpected or sudden deaths (please see page 10- Implications for practice).

VERSION 2 – REVIEW

**REVIEWER NAME**	Garstang, Joanna
**REVIEWER AFFILIATION**	University of Birmingham College of Medical and Dental Sciences, School of Nursing and Midwifery
**REVIEWER CONFLICT OF INTEREST**	No Conflict of Interest
**AI DISCLOSURE**	No, I have not used AI
**DATE REVIEW RETURNED**	04-Feb-2026

**GENERAL COMMENTS**	Thank you for making the revisions to this paper. I think it is now an excellent paper and deserves to be published. It is a great piece of research.
